# Supplementary material for: The parent-of-origin lncRNA MISSEN regulates rice endosperm development
Source: Nat Commun. 2021 Nov 11;12:6525. doi: 10.1038/s41467-021-26795-7 (PMC8585977; doi:10.1038/s41467-021-26795-7)
Supplement: Supplementary file 1 — Supplementary Information [file 41467_2021_26795_MOESM1_ESM.pdf]

Supplementary Materials for

The parent-of-origin lncRNA *MISSEN* regulates rice endosperm development

Yan-Fei Zhou[1#], Yu-Chan Zhang[1#], Yu-Meng Sun[1#], Yang Yu[1], Meng-Qi Lei[1], Yu-Wei Yang[1], Jian-Ping Lian[1], Yan-Zhao Feng[1], Zhi Zhang[1] , Lu Yang[1], Rui-Rui He[1], Jia-Hui Huang[1], Yu Cheng[1], Yu-Wei Liu and Yue-Qin Chen[1,2]*

Correspondence to: lsscyq@mail.sysu.edu.cn to YQC

This PDF file includes:

Supplementary Figures 1 to 7
Supplementary Tables 1 to 2

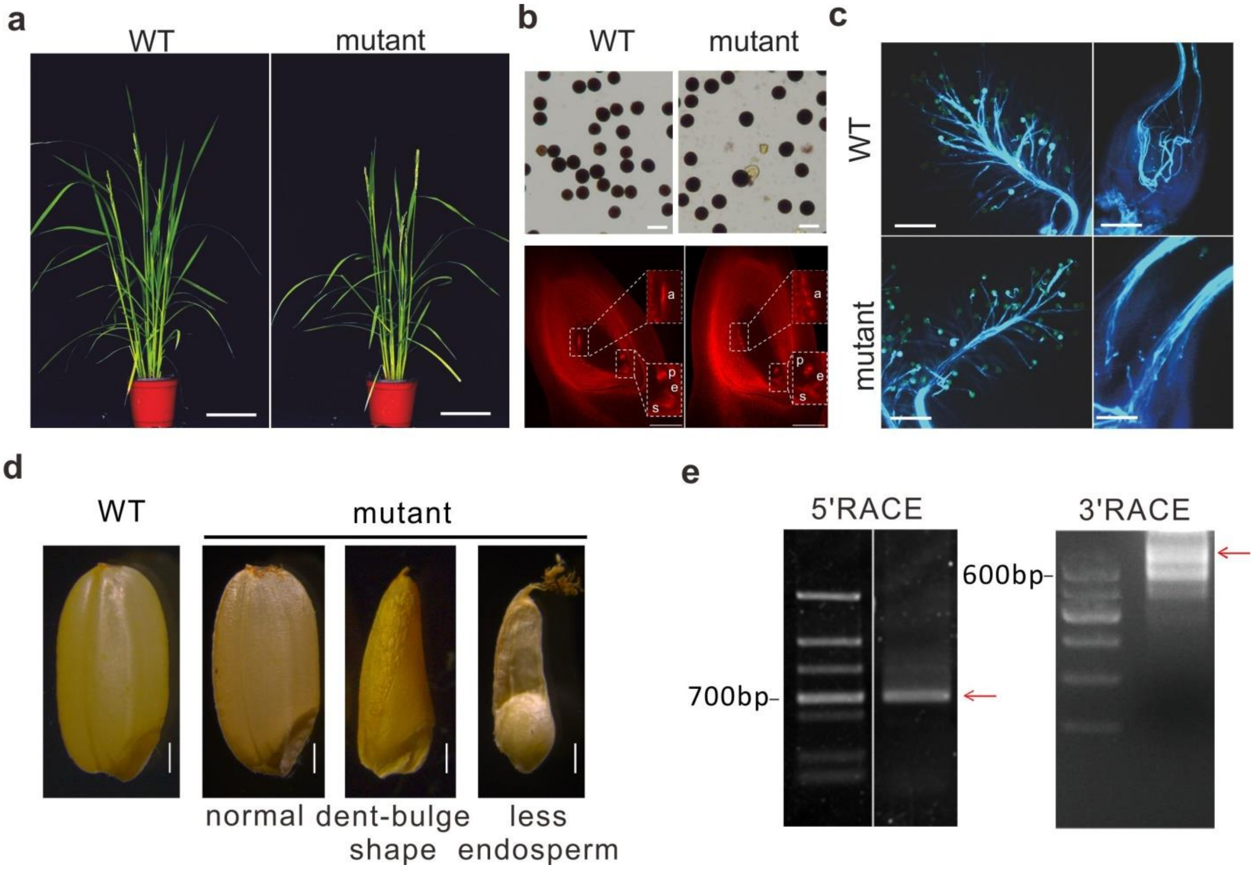

Supplementary Fig. 1. Phenotypes of T-DNA insertion mutant plants. **a** The WT and the T-DNA insertion mutant. Scale bars, 15cm; **b** The pollen grains and ovaries of the WT and the mutant. Scale bars, 50μm. The experiment was repeated 3 times with similar results and one representative result was shown; **c** *In vivo* pollen germination assay of the WT and the T-DNA insertion mutant. Scale bars, 500μm. The experiment was repeated 3 times with similar results and one representative result was shown; **d** The morphology of seeds with a "dent-bulge shape" or less-endosperm. Scale bars, 1mm; **e** Rapid amplification of cDNA ends to experimentally validate the 5′ and 3′ ends of *MISSEN*. The experiment was repeated 3 times with similar results and one representative result was shown.

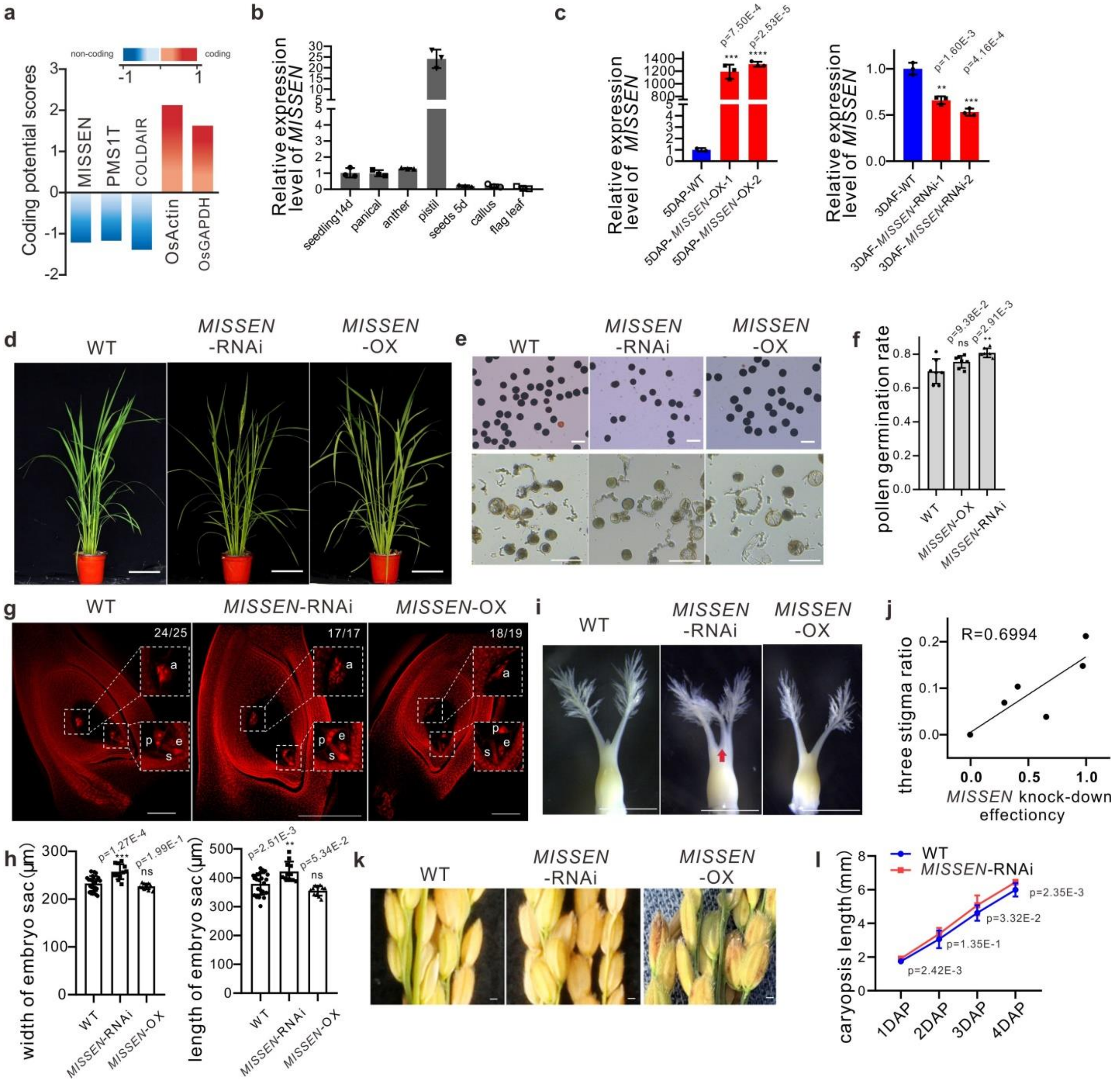

Supplementary Fig. 2. Phenotypes of different transgenic plants. **a** Coding potential score of *MISSEN*; **b** The expression pattern of *MISSEN* in different tissues. Values are the means ± SD (*n* = 3 replicates, normalized against *ACTIN2*); **c** Relative expression level of *MISSEN* in *MISSEN*-OX and *MISSEN*-RNAi plants. Values are the means ± SD (*n* = 3 replicates, normalized against *ACTIN2*). **d** The WT, *MISSEN*-RNAi, and *MISSEN*-OX plants. Scale bars, 15cm; **e** The pollen grains and pollen germination of WT, *MISSEN*-RNAi, and *MISSEN*-OX plants. Scale bars, 50μm for pollen grains and 100μm for germination; **f** The pollen germination ratio in WT, mutant, *MISSEN*-RNAi, and *MISSEN*-OX plants.Values are the means ± SD (*n* = 7). **g-i** The

mature ovaries (**g**) and stigmas (**i**) in WT, *MISSEN*-RNAi, and *MISSEN*-OX plants. Scale bars, 100μm for ovaries and 1mm for stigmas. The statistics of width and length of embryo sac in WT, *MISSEN*-RNAi, and *MISSEN*-OX plants were shown in **h**. The numbers in the images indicate the proportion of samples that exhibited the phenotype. Values are the means ± SD (*n* =24, 11, 12 for width and *n* = 24, 11, 11 for length); **j** Correlation analysis between RNAi interference level and three stigma ratio in *MISSEN*-RNAi plants; **k** The panicles of WT, *MISSEN*-RNAi, and *MISSEN*-OX plants at 21 DAP. Scale bars, 1mm; **l** The changes of the caryopsis length during 1-4 days after pollination in WT and *MISSEN*-RNAi plants. Values are the means ± SD (*n* =9, 11 at 1DAP; *n* =12, 9 at 2DAP; *n* =15, 12 at 3DAP; *n* =15, 10 at 4DAP). Significant differences were identified at the 5% (*), 1% (**), 0.1% (***) and less than 0.01% (****) probability levels using two-tailed paired t-test.

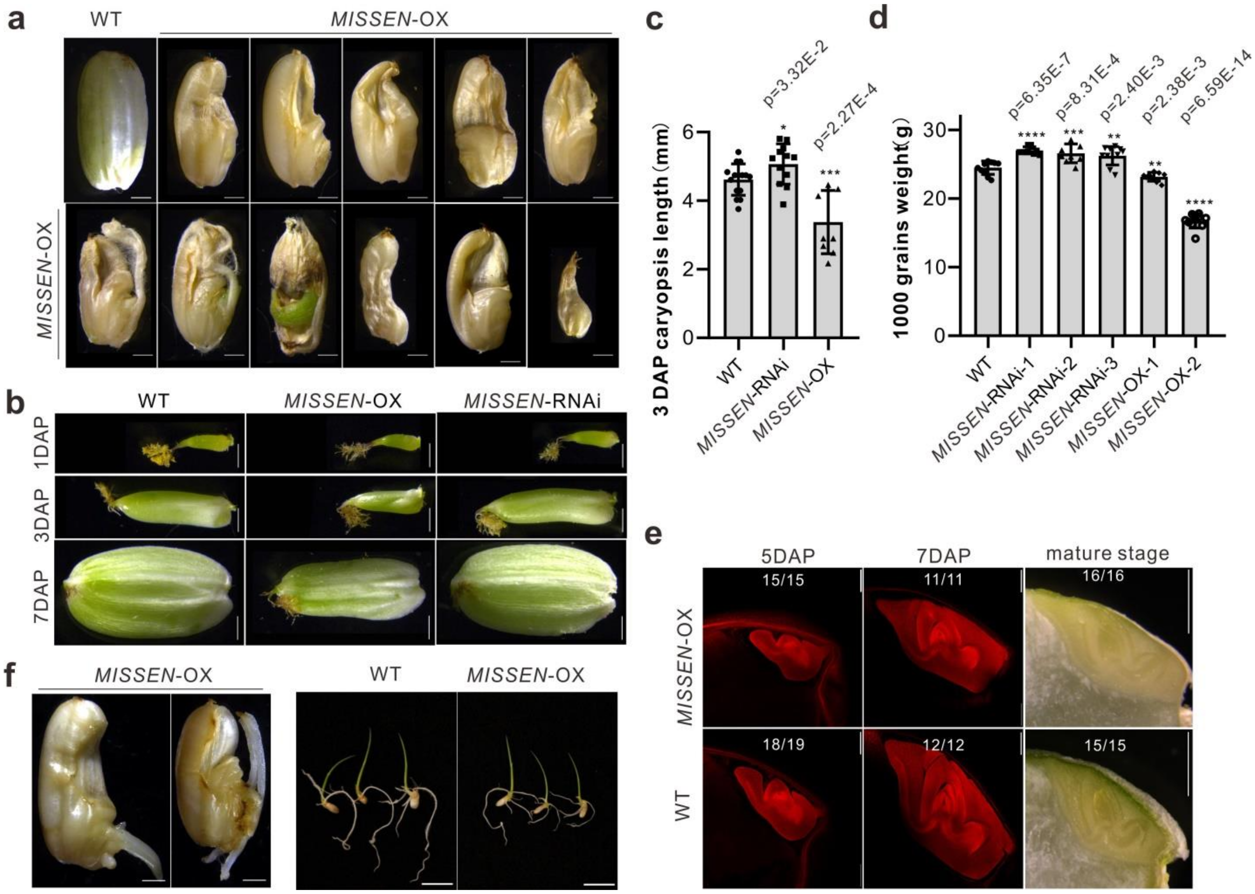

Supplementary Fig. 3. The seeds of different transgenic plants. **a** The seeds of *MISSEN*-OX plants at the mature stage. Scale bars, 1mm; **b** The developing caryopses of WT, *MISSEN*-OX, and *MISSEN*-RNAi plants. Scale bars, 1mm; **c** The caryopsis length of WT, *MISSEN*-RNAi, and *MISSEN*-OX plants at 3DAP. Values are the means ± SD (*n* =15, 12, 9 caryopsis); **d** The 1000 grains weight of mature seeds in WT, *MISSEN*-RNAi-1, *MISSEN*-RNAi-2, *MISSEN*-RNAi-3, *MISSEN*-OX-1 and *MISSEN*-OX-2 plants. Values are the means ± SD (*n* =11, 10, 9, 11, 9, 11 plants); **e** The phenotype of embryo at 5DAP, 7DAP and mature stage in WT and *MISSEN*-OX plants. Scale bars of eosin B-staining and freehand section are 100μm and 1mm respectively. The numbers in the images indicate the proportion of samples that exhibited the phenotype; **f** Germination of the abnormal *MISSEN*-OX seeds. Scale bars, 1mm for seeds and 1cm for seedlings. Significant differences were identified at the 5% (*), 1% (**), 0.1% (***) and less than 0.01% (****) probability levels using two-tailed paired t-test.

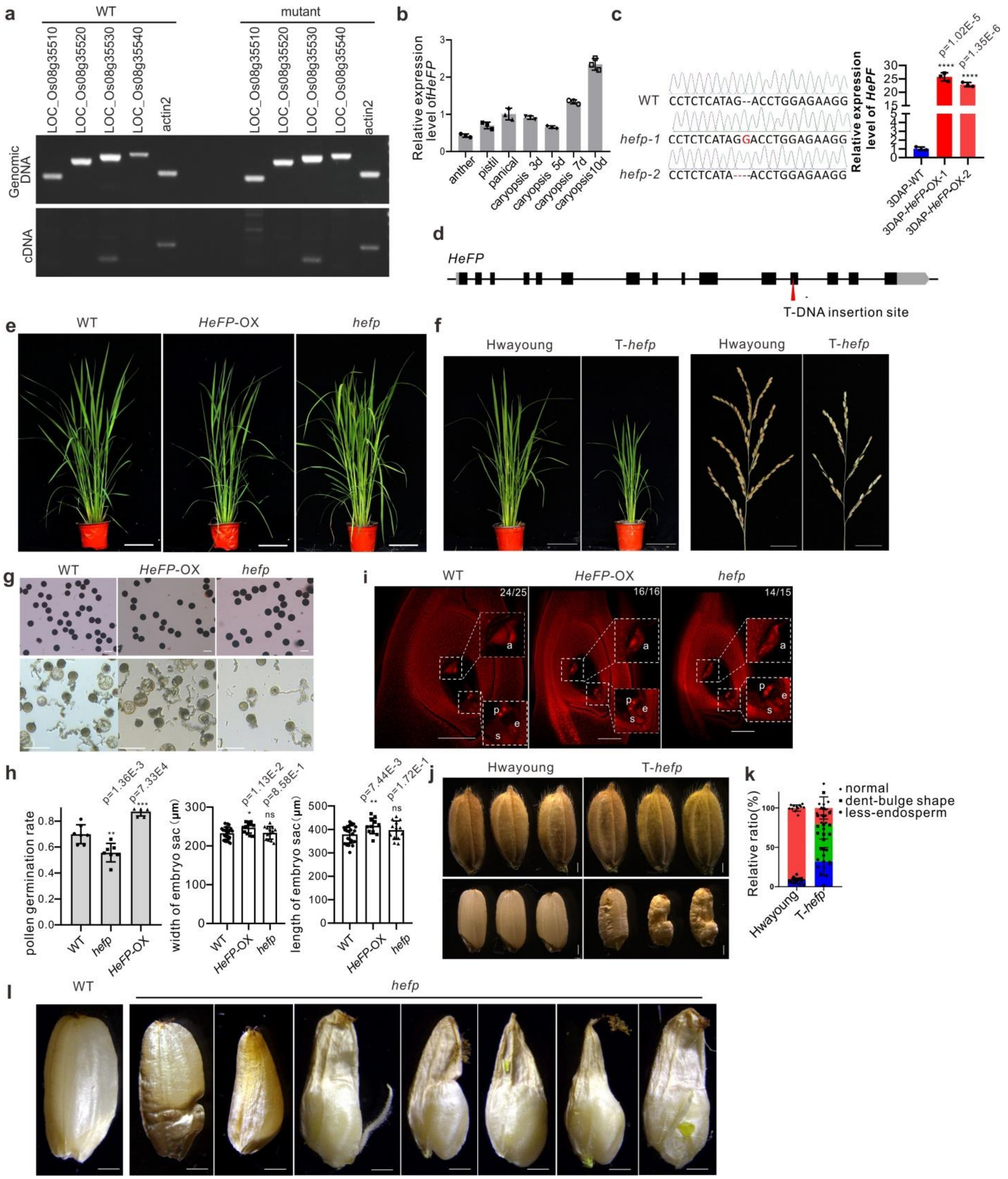

Supplementary Fig. 4. The expression of genes near *MISSEN* in the T-DNA insertion mutant, and the phenotype analysis of *hefp* and *HeFP*-OX plants. **a** The expression of genes near *MISSEN* in the T-DNA insertion mutant; **b** The expression pattern of *HeFP* in different tissues.

Values are the means ± SD (*n* = 3 replicates, normalized against *ACTIN2*); **c** The genotypes of *hefp* plants and the relative expression level of *HeFP* mRNAs in the *HeFP*-OX plants. Values are the means ± SD (*n* = 3 replicates, normalized against *ACTIN2*); **d** The gene structure of the *HeFP*. The T-DNA insertion site of the mutant is indicated by the red arrow; **e** The WT and transgenic plants. Scale bars, 15cm; **f** Phenotype and panicle of T-*hefp* plants. Scale bars, 15cm for plants and 3cm for panicle; **g** The pollen grains and pollen germination of WT, *HeFP*-OX and *hefp* plants. Scale bars, 50μm for pollen grains and 100μm for germination; **h** The pollen germination ratio in WT, *hefp* and *HeFP*-OX plants. Values are the means ± SD (*n* = 7, 6, 5). The statistics of width and length of embryo sac in WT, *HeFP*-OX and *hefp* plants. Values are the means ± SD (*n* =24, 12, 13); **i** The mature ovaries in WT, *HeFP*-OX and *hefp* plants. Scale bars, 100μm; **j** The seeds of T-*hefp*. Scale bars, 1mm. The numbers in the images indicate the proportion of samples that exhibited the phenotype; **k** The ratio of abnormal seeds in T-*hefp* plants. Values are the means ± SD (n = 13 plants); **l** The seeds of *hefp* plants at the mature stage. Scale bars, 1mm. Significant differences were identified at the 5% (*), 1% (**), 0.1% (***) and less than 0.01% (****) probability levels using two-tailed paired t-test.

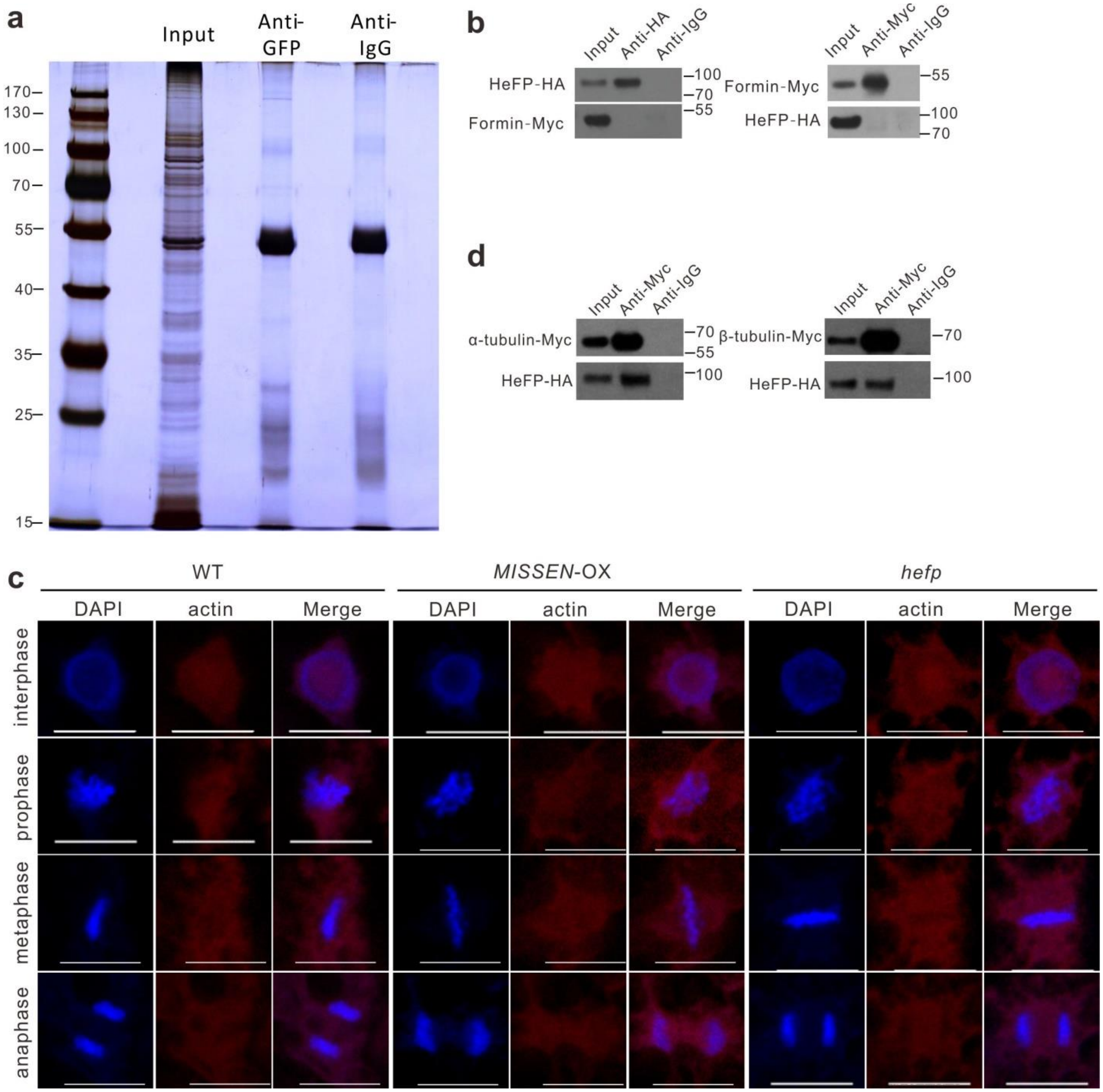

Supplementary Fig. 5. Identification of interacting proteins of HeFP. **a** Co-IP assay of HeFP. The experiment was repeated 3 times with similar results and one representative result was shown; **b** *In vitro* binding assays between HeFP and formin. The experiment was repeated 3 times with similar results and one representative result was shown; **c** Microfilament arrays were visualized by immunostaining with TRITC Phalloidin during mitosis in WT, *MISSEN*-OX and *hefp* endosperm cells at 3 DAP. Microfilaments are colored red, and nuclei or chromosomes are colored blue. Scale bars, 10μm. The experiment was repeated 3 times with similar results and

one representative result was shown; **d** *In vitro* binding assays between HeFP and α-tubulin, β-tubulin. The experiment was repeated 3 times with similar results and one representative result was shown.

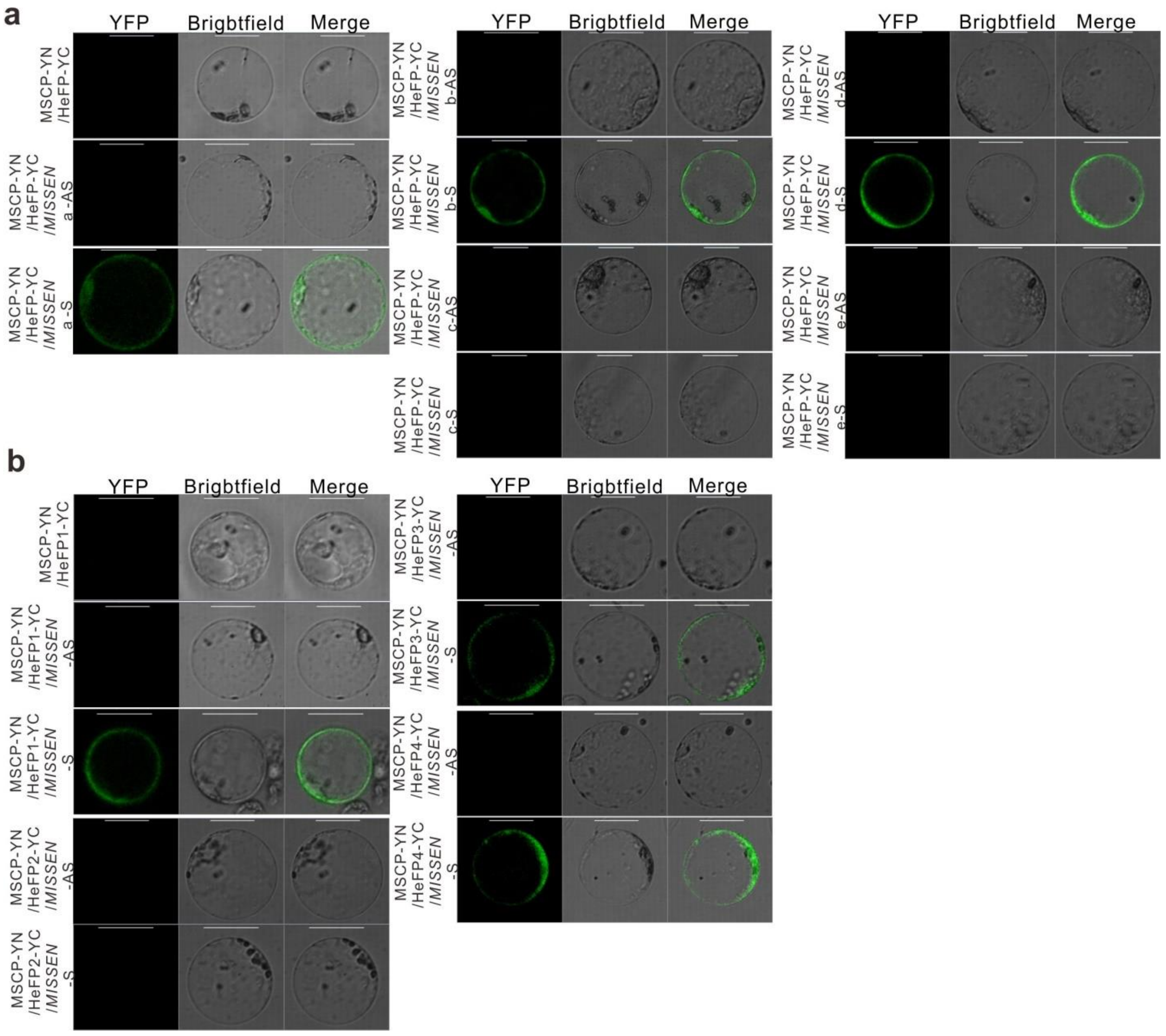

Supplementary Fig. 6. TriFC assay of truncated *MISSEN* and HeFP mutants. **a** TriFC assay of *MISSEN* fragments and HeFP. The antisense sequences of *MISSEN* fragments as control. Scale bars, 20μm. The experiment was repeated 3 times with similar results and one representative result was shown; **b** TriFC assay of *MISSEN* and truncated HeFP sections. The antisense sequences of *MISSEN* as control. Scale bars, 20μm. The experiment was repeated 3 times with similar results and one representative result was shown.

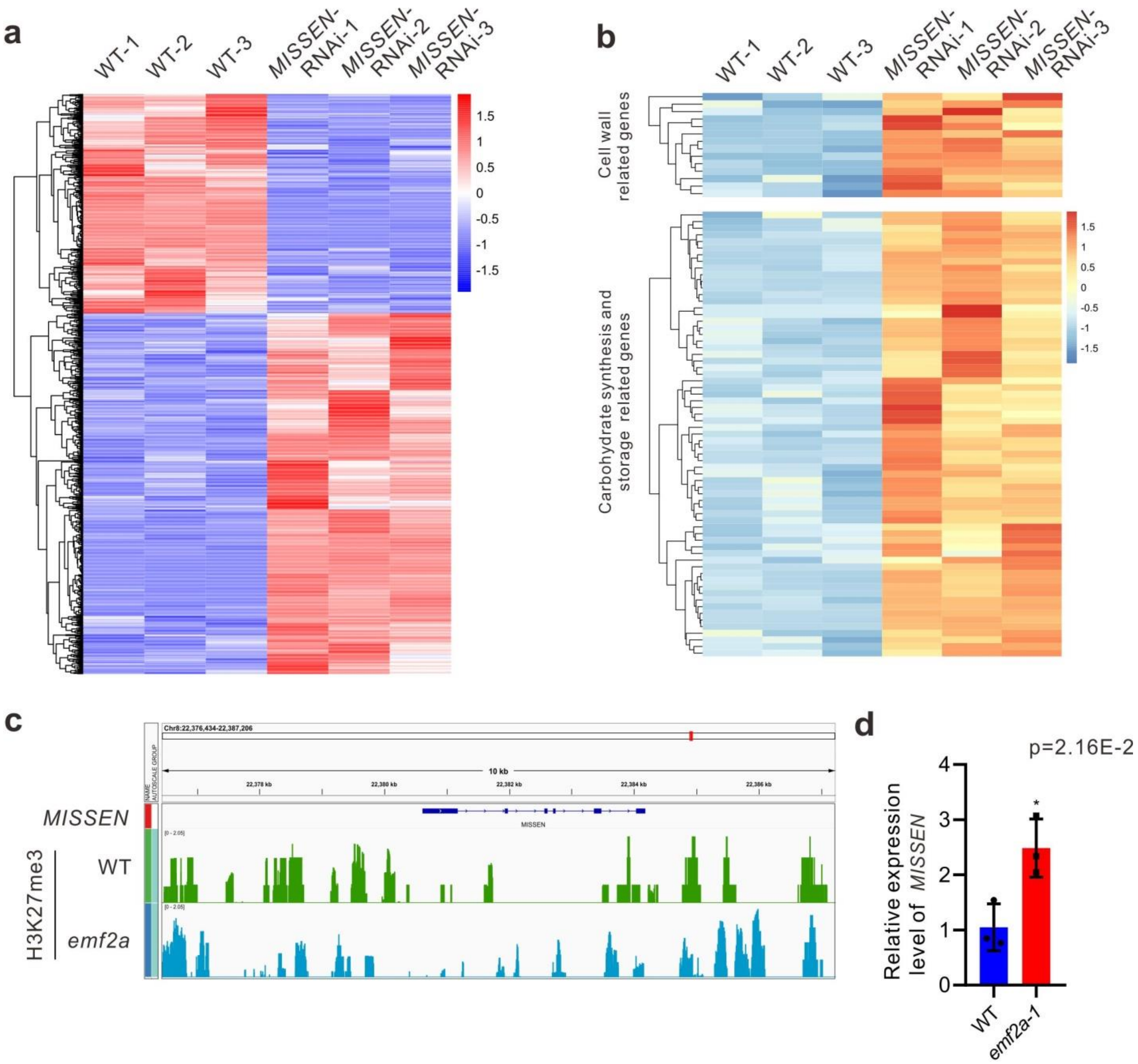

Supplementary Fig. 7. Knockdown of *MISSEN* with RNAi promotes endosperm development and *MISSEN* expression is repressed by H3K27me3. **a** Heatmap of the expression of differentially expressed genes in caryopsis of *MISSEN*-RNAi compared to WT at 7DAP; **b** The effects of *MISSEN*-RNAi on the expression of cell wall-related genes (up panel) and carbohydrate synthesis and storage-related genes (down panel); **c** The level of H3K27me3 modification on -2.5kb upstream of *MISSEN* in WT and *emf2a* endosperm; **d** Relative expression level of *MISSEN* in WT and *emf2a*. Values are the means ± SD (n = 3 replicates, normalized against *ACTIN2*). Significant differences were identified at the 5% (*), 1% (**), 0.1% (***) and less than 0.01% (****) probability levels using two-tailed paired t-test.

Supplementary Table 1. Information of primers used in this study.

**Name**	**Forward Primer**	**Reverse Primer**
q-actin2	TCTTACGGAGGCTCCACTTAAC	TCCACTAGCATAGAGGGAAAGC
hefp	GCCGCATGGCGCCTCTCATAGACC	AAACGGTCTATGAGAGGCGCCATG
g-*HeFP*	AGATGAGGCTCGCGAAGCGG	TGGAAGCACGTCGCCCTTGT
HeFP-OX	CGGGATCCGCGGGGCGGAGTGGCG	CGGAATTCGCTCTGGTATTCTGATGCACTCAAGTACTC
q-*HeFP*	ACACACGATGTGGTTGCTCT	CAAGGCGGAGAGGGCTATTT
q-*MISSEN*-X1	ATCTTGCAGCCTCTATCGGTG	GGCAGGAGCACGATGTGT
q-*MISSEN*-X2	AGGGTGGTTGTCTTGCGTCT	GCTGGAGATGCAGATCCTCAA
q-*MISSEN*-X3	CCCCTACTGCCGTCTTCTTG	ATTCTGAAATGCCTGATGTTGC
q-*MISSEN*-X3/4	AATGGCAATCAGTAGGTAGAGACG	AATCCGTTCAAGTCCCTGTAGC
LOC_Os08g35510	TACTCGGGCTGATCAAACGC	CGCCGATGAGGTTGAAGTTG
LOC_Os08g35520	TCCGACCCATACTGGCAAAC	TCACCATTCCTGGATCATAATCAC
LOC_Os08g35530	GTCTGTTGATAAGGTAGGTGGCTGT	AGCATCCAGTGCGACAACG
LOC_Os08g35540	CCACCGCTCCTTTCTCTGTT	AAAGTAGTTGTCGGAGATAACGTGA
MISSEN-OX	CGGGATCCCCGCGCACGCAGAAAA	GGACTAGTACTCAAATCCAATGTTCTAACAAGC
c-*MISSEN*	GTGTGGGTTTAAATGCTGG	AAACCTGCGCCAGCATTTAAA

	CGCAG	CCC
g-*MISSEN*	CACAGAAAACGGAGCAGTTCATCAGC	GACGCAAGACAACCACCCTCAGC
RNAi-*MISSEN*-S	CGGGATCCCCGCGCACGCAGAAAA	CGAGCTCCCACGTACGTACGTACGTACGTA
RNAi-*MISSEN*-AS	CGACGCGTCCGCGCACGCAGAAAA	TGCACTGCAGCCACGTACGTACGTACGTACGTA

Supplementary Table 2. Information of antibodies used in this study.

Antibody	corporation	Cat.No.	Antibody	corporation	Cat.No.
anti-tubulin	Abcam	Ab7291	anti-HA	CST	#3724
anti-Myc-tag	Clonetech	631 206	anti-GFP	TransGen	HT801-01
anti-GST	CST	#2622	anti-HA	Sigma	H9658
anti-Myc-tag	Proteintech	16286-1-AP	anti-GAPDH	BPI	AbP80006-A-SE
anti-Flag	Sigma	F1804	Anti-H3	Abcam	Ab1791
Anti-H3K27me3	Abcam	6002	Goat Anti-Mouse IgG(H+L) AF488	Trans	HS231-01
