# Supplementary material for: The parent-of-origin lncRNA MISSEN regulates rice endosperm development
Source: Nat Commun. 2021 Nov 11;12:6525. doi: 10.1038/s41467-021-26795-7 (PMC8585977; doi:10.1038/s41467-021-26795-7)
Supplement: Supplementary file 3 — Description of Additional Supplementary Files [file 41467_2021_26795_MOESM3_ESM.pdf]

Description of Additional Supplementary Files

Supplementary Data 1: Proteins identified in specific bands by MS may interact with MISSEN.

Supplementary Data 2: Proteins identified in specific bands by MS may interact with HePF.

Supplementary Data 3: The GO terms of the DEGs between WT and MISSEN-RNAi 7DAG caryopsis.

Supplementary Data 4: FPKM of carbohydrate and cell wall related genes.
