# Supplementary material for: The parent-of-origin lncRNA MISSEN regulates rice endosperm development
Source: Nat Commun. 2021 Nov 11;12:6525. doi: 10.1038/s41467-021-26795-7 (PMC8585977; doi:10.1038/s41467-021-26795-7)
Supplement: Supplementary file 8 — Reporting Summary [file 41467_2021_26795_MOESM8_ESM.pdf]

nature portfolio

Corresponding author(s): Yue-Qin Chen

Last updated by author(s): Oct 13, 2021

Reporting Summary

Nature Portfolio wishes to improve the reproducibility of the work that we publish. This form provides structure for consistency and transparency in reporting. For further information on Nature Portfolio policies, see our Editorial Policies and the Editorial Policy Checklist.

Statistics

For all statistical analyses, confirm that the following items are present in the figure legend, table legend, main text, or Methods section.

n/a	Confirmed	
☐	☒	The exact sample size (n) for each experimental group/condition, given as a discrete number and unit of measurement
☐	☒	A statement on whether measurements were taken from distinct samples or whether the same sample was measured repeatedly
☐	☒	The statistical test(s) used AND whether they are one- or two-sided<br>*Only common tests should be described solely by name; describe more complex techniques in the Methods section.*
☒	☐	A description of all covariates tested
☒	☐	A description of any assumptions or corrections, such as tests of normality and adjustment for multiple comparisons
☐	☒	A full description of the statistical parameters including central tendency (e.g. means) or other basic estimates (e.g. regression coefficient) AND variation (e.g. standard deviation) or associated estimates of uncertainty (e.g. confidence intervals)
☐	☒	For null hypothesis testing, the test statistic (e.g. F, t, r) with confidence intervals, effect sizes, degrees of freedom and P value noted<br>*Give P values as exact values whenever suitable.*
☒	☐	For Bayesian analysis, information on the choice of priors and Markov chain Monte Carlo settings
☒	☐	For hierarchical and complex designs, identification of the appropriate level for tests and full reporting of outcomes
☒	☐	Estimates of effect sizes (e.g. Cohen's d, Pearson's r), indicating how they were calculated

Our web collection on statistics for biologists contains articles on many of the points above.

Software and code

Policy information about availability of computer code

Data collection	Oryza sativa genome assembly RGAP 7.0 was used throughout this study and was downloaded from http://rice.plantbiology.msu.edu/.
Data analysis	For the analysis of transcriptome sequencing, all clean reads were aligned to the reference genome Oryza sativa sp. japonica cv. Nipponbare (RGAP 7) using STAR v2.7.5c. The aligned read counts were obtained from alignment files using featureCounts v2.0.1. Differentially expressed genes analysis was conducted using the R package DESeq2 v1.32.0 . The Gene Ontology enrichment analysis of the differentially expressed genes was performed on the AgriGOv2 website (http://systemsbiology.cau.edu.cn/agriGOv2/). Heatmaps were ploted using the R package Pheatmap v1.0.12.<br>For chip-seq analysis, all clean reads were aligned to the reference genome Oryza sativa sp. japonica cv. Nipponbare (RGAP 7) using bowtie2 version 2.4.1. Multi-mapped reads and PCR duplicated reads were discarded using samtools versions 1.9. peaks were called by the callpeak function of MACS2 v2.1.2 with options [-broad –broad-cutoff 0.1 -g 3.7e8].

For manuscripts utilizing custom algorithms or software that are central to the research but not yet described in published literature, software must be made available to editors and reviewers. We strongly encourage code deposition in a community repository (e.g. GitHub). See the Nature Portfolio guidelines for submitting code & software for further information.

Data

Policy information about availability of data

All manuscripts must include a data availability statement. This statement should provide the following information, where applicable:

- Accession codes, unique identifiers, or web links for publicly available datasets
- A description of any restrictions on data availability
- For clinical datasets or third party data, please ensure that the statement adheres to our policy

The source data for Figs. 1–7 and Supplementary Figs.2–5, 7 are provided as a Source Data file. The transcriptome datasets are uploaded to the NCBI SRA database (SRA Accession No. PRJNA765401 [https://www.ncbi.nlm.nih.gov/sra/PRJNA765401]). The H3K27me3 Chip-seq data was obtained from publicly available sources (DDBJ: DRA010700 [DRA, https://www.ddbj.nig.ac.jp/dra/index-e.html]).

Field-specific reporting

Please select the one below that is the best fit for your research. If you are not sure, read the appropriate sections before making your selection.

☒ Life sciences ☐ Behavioural & social sciences ☐ Ecological, evolutionary & environmental sciences

For a reference copy of the document with all sections, see nature.com/documents/nr-reporting-summary-flat.pdf

Life sciences study design

All studies must disclose on these points even when the disclosure is negative.

Sample size	10 or more rice plants are usually sufficient for gene expression level analysis and phenotype analysis to eliminate individual difference. In this study, 15 or more individual plants of each transgenic line were obtained and subjected to statistical analyses.
Data exclusions	All the samples were chosen randomly that there is no exclusion criteria.
Replication	At least two independent transgenic lines were used in each analysis. ALL the gene expression level analysis and phenotype analysis have more than three replicates.
Randomization	All samples were allocated into experimental groups randomly.
Blinding	The investigators were blinded to group allocation during data collection and analysis.

Reporting for specific materials, systems and methods

We require information from authors about some types of materials, experimental systems and methods used in many studies. Here, indicate whether each material, system or method listed is relevant to your study. If you are not sure if a list item applies to your research, read the appropriate section before selecting a response.

Materials & experimental systems

n/a	Involved in the study	
☐	☒	Antibodies
☒	☐	Eukaryotic cell lines
☒	☐	Palaeontology and archaeology
☒	☐	Animals and other organisms
☒	☐	Human research participants
☒	☐	Clinical data
☒	☐	Dual use research of concern

Methods

n/a	Involved in the study	
☒	☐	ChIP-seq
☒	☐	Flow cytometry
☒	☐	MRI-based neuroimaging

Antibodies

Antibodies used

anti-Tubulin (Ab7291, Abcam, mouse),
anti-HA (#3724, CST, rabbit),
anti-Myc-tag (631 206, Clonetech, mouse),
anti-GFP (HT801-01, TransGen, mouse),
anti-GST(#2622, CST, rabbit),
anti-HA(H9658, Sigma, rabbit),
anti-Myc-tag (16286-1-AP, Proteintech, rabbit),
anti-GAPDH (AbP80006-A-SE, BPI, rabbit),
anti-H3(Ab1791, Abcam, rabbit),

	Anti-H3K27me3(Ab6002, Abcam, mouse)<br>Goat Anti-Mouse IgG(H+L) AF488 HS231-01Trans
Validation	All antibodies were validated by the manufacturers and in publications lists one the manufacturers websites.
